# Preventive HIPEC in combination with perioperative FLOT versus FLOT alone for resectable diffuse type gastric and gastroesophageal junction type II/III adenocarcinoma – the phase III “PREVENT”- (FLOT9) trial of the AIO /CAOGI /ACO

**DOI:** 10.1186/s12885-021-08872-8

**Published:** 2021-10-29

**Authors:** Thorsten O. Götze, Pompiliu Piso, Sylvie Lorenzen, Ulli S. Bankstahl, Claudia Pauligk, Moustafa Elshafei, Giuseppe Amato, Daniel Reim, Wolf O. Bechstein, Alfred Königsrainer, Stefan P. Mönig, Beate Rau, Matthias Schwarzbach, Salah-Eddin Al-Batran

**Affiliations:** 1 Institute of Clinical Cancer Research (IKF) at Krankenhaus Nordwest, UCT-University Cancer Center, Frankfurt, Germany; 2 Institut für Klinische Krebsforschung IKF GmbH am Krankenhaus Nordwest, Frankfurt, Germany; 3 Department for General and Visceral Surgery, Hospital Barmherzige Brueder, University of Regensburg, Regensburg, Germany; 4 Third Department of Internal Medicine (Hematology/Medical Oncology), Klinikum Rechts der Isar, Technische Universitat Munchen, Munich, Germany; 5 Bariatrische und Metabolische Chirurgie, Krankenhaus Nordwest, Frankfurt, Germany; 6 Department of General Surgery and Emergency, University of Palermo, Palermo, Italy; 7 Klinik und Poliklinik für Chirurgie, Klinikum rechts der Isar, Technische Universität München, Munich, Germany; 8 Department of General and Visceral Surgery, University Hospital Frankfurt, Frankfurt, Germany; 9 Department of General-, Visceral Surgery and Transplantation, University Hospital Tübingen, Tübingen, Germany; 10 Service de Chirurgie viscérale, Hôpitaux Universitaires de Genève, Geneva, Switzerland; 11 Department of Surgery, Campus Charité Mitte, Campus Virchow-Klinikum CCM/CVK, Berlin, Germany; 12 Clinic for General, Visceral, Vascular and Thoracic Surgery, Klinikum Frankfurt Höchst, Frankfurt, Germany

**Keywords:** Gastric cancer, Gastroesophageal junction cancer, Lauren- classification, Signet ring cells, HIPEC, Hyperthermic intraperitoneal chemotherapy, FLOT- regimen, Gastrectomy, Quality of life, Cisplatin, Peritoneal carcinomatosis

## Abstract

**Background:**

The main reason for treatment failure after curative surgical resection of gastric cancer is intra-abdominal spread, with 40–50% peritoneal seeding as primary localization of recurrence. Peritoneal relapse is seen in 60–70% of tumors of diffuse type, compared to only 20–30% of intestinal type.

Hyperthermic IntraPEritoneal Chemoperfusion (HIPEC) is an increasingly used therapy method for patients with peritoneal metastases. The preventive use of HIPEC could represent an elegant approach for patients (pts) before macroscopic peritoneal seeding, since pts. with operable disease are fit and may have potential risk of microscopic involvement, thus having a theoretical chance of cure with HIPEC even without the need for cytoreduction.

No results from a PCRT from the Western hemisphere have yet been published.

**Methods:**

This is a multicenter, randomized, controlled, open-label study including a total of 200 pts. with localized and locally advanced diffuse or mixed type (Laurens’s classification) adenocarcinoma of the stomach and Type II/III GEJ.

All enrolled pts. will have received 3–6 pre-operative cycles of biweekly FLOT (Docetaxel 50 mg/m^2^; Oxaliplatin 85 mg/m^2^; Leucovorin 200 mg/m^2^; 5-FU 2600 mg/m^2^, q2wk).

Pts will be randomized 1:1 to receive surgery only and postoperative FLOT (control arm) or surgery + intraoperative HIPEC (cisplatin 75 mg/m^2^ solution administered at a temperature of 42 °C for 90 min) and postoperative FLOT (experimental arm). Surgery is carried out as gastrectomy or transhiatal extended gastrectomy. Primary endpoint is PFS/DFS, major secondary endpoints are OS, rate of pts. with peritoneal relapse at 2 and 3 years, perioperative morbidity/mortality and quality of life.

The trial starts with a safety run-in phase. After 20 pts. had curatively intended resection in Arm B, an interim safety analysis is performed.

Recruitment has already started and first patient in was on January 18th, 2021.

**Discussion:**

If the PREVENT concept proves to be effective, this could potentially lead to a new standard of therapy. On the contrary, if the outcome is negative, pts. with gastric cancer and no peritoneal involvement will not be treated with HIPEC during surgery.

**Trial registration:**

The study is registered on June 25th, 2020 under ClinicalTrials.gov Identifier: NCT04447352; EudraCT: 2017-003832-35.

## Background

The main reason for treatment failure after curative surgical resection of gastric cancer is intra-abdominal spread. Local recurrence, retroperitoneal lymph node metastases, peritoneal seeding and liver metastases are observed in about 90% of patients having tumor progression. Most recurrences are reported within 18 months from the primary surgical resection. In 40–50% of these cases, a peritoneal seeding is the primary localization of recurrence. The likelihood for a peritoneal relapse is even much more common in the diffuse type gastric cancer according to Lauren’s classification, and ranges between 60 and 70% [1–5]. On the other hand, intestinal type tumors tend to spread via hematogenous routes and show only a peritoneal seeding rate of 20–30%.

Therefore, the outcome of diffuse type respectively signet-ring cell gastric cancer including the mixed types acc. to Lauren, remains unsatisfactory. Signet ring cell gastric cancer is associated with younger age; usually affects the corpus of the stomach and presents rapid relapse and worse prognosis compared with the intestinal type. Moreover, the response of peritoneal metastases to systemic chemotherapy is poor, mainly due to the presence of a so called “plasma-peritoneal barrier” which isolates the peritoneal cavity from the effects of intravenous chemotherapy [6].

Taken together, considerable investigation is still required to improve perioperative protocols, particularly the intra-operative component, in this aggressive subgroup of gastric cancer.

FLOT, a docetaxel-based triplet combination consisting of 5-FU, leucovorin, oxaliplatin and docetaxel, (Docetaxel 50 mg/m^2^ in 250 ml NaCl 0.9%, iv over 1 h; Oxaliplatin 85 mg/m^2^ in 500 ml G5%, iv over 2 h; Leucovorin 200 mg/m^2^ in 250 ml NaCl 0.9%, iv over 30 min; 5-FU 2600 mg/m^2^, iv over 24 h, q2wk) [7, 8] is one of the most intensively evaluated regimens for gastric and GEJ adenocarcinoma. It has been evaluated in the metastatic setting [9], in the limited metastatic setting [10], in elderly patients [11] and in operable patients [7]. The AIO FLOT4 phase II/III study has evaluated FLOT versus Epirubicin, Cisplatin and 5-FU (ECF) as well Epirubicin, Cisplatin and Xeloda (ECX) (*n* = 716). The phase II part of the randomized phase II/III FLOT4 trial regarding histopathological regression [7] comprised 300 patients, of whom 265 patients were evaluable on an intent-to-treat basis. FLOT was associated with significantly higher proportions of patients achieving pathological complete regression than ECF/ECX (20 [16%; 95% CI 10–23] of 128 patients vs 8 [6%; 3–11] of 137 patients; *p* = 0.02). Also the rate of complete or subtotal regression (TRG1a/b) was significantly higher with FLOT (47 [37%] of 128 vs. 31 [23%] of 137, p = 0.02). The differences were more pronounced in intestinal type tumors. Despite the problems of systemic chemotherapy in diffuse type gastric cancers, FLOT was able the show efficacy in this type of gastric cancers according to the phase III data of the FLOT4 trial [8]. FLOT is regarded a standard chemotherapy regimen for gastric cancer in the perioperative setting. So FLOT is also regarded as the most effective backbone protocol for the current multimodal trial for patients suffering from diffuse type (acc. to Lauren) gastric or GEJ adenocarcinoma.

Hyperthermic Intraperitoneal Chemoperfusion (HIPEC) is an increasingly used therapy method for patients with peritoneal metastases. Although some evidence exists on its efficacy for selected disease entities it could not become standard of care due to lack of randomized trials. Moreover, patients with macroscopic involvement of the peritoneal cavity are less likely to be cured, leading many physicians to avoid burdensome therapy approaches including HIPEC for these patients. In contrast, the preventive use of HIPEC could represent a more elegant approach, since patients with operable disease are fit and have no macroscopic visible peritoneal involvement, thus having a theoretical chance of cure. This would justify more intense therapy regimen. Unfortunately, current data regarding HIPEC- procedure in different tumor entities are contradictory.

Among patients with a stage III epithelial ovarian cancer, the addition of cisplatin based HIPEC to cytoreductive surgery resulted in an improvement of recurrence-free and overall survival compared to surgery alone and established the role of HIPEC in ovarian cancer entity as more or less a standard procedure in stage III patients based on phase III data [12].

On the other hand, in colorectal cancer data regarding intraperitoneal therapy with HIPEC are currently not as promising as in the ovarian entity. PROPHYLOCHIP–PRODIGE 15 showed that systematic second-look surgery including oxaliplatin based HIPEC-therapy did not improve disease-free survival compared with standard surveillance only [13]. In addition, PRODIGE- 7 showed also no benefit in overall survival after adding HIPEC to cytoreductive surgery with this combination in colorectal cancer with an existing peritoneal seeding and a Peritoneal Cancer Index of 25 or less and the intent for a curative approach in a metastatic disease [14].

A registry study considering 152 patients [15] could show that in cytoreductive surgeries for peritoneal metastases in small bowel adenocarcinoma combined with the HIPEC procedure achieved prolonged survival for selected patients with acceptable morbidity and mortality.

Nevertheless, HIPEC- procedure is still an established procedure according e.g. to the S3- guidelines in Germany [16] in metastatic colorectal cancer patients, but based on the French data a matter of debate [17].

Glehen et al. published a report on the French experience in the curative treatment of gastric peritoneal carcinomatosis [18], in a multi-institutional study of 159 patients treated by cytoreductive surgery combined with perioperative intraperitoneal chemotherapy. The therapeutic approach combining cytoreductive surgery with intraperitoneal chemotherapy for patients with gastric carcinomatosis achieved a long-term survival in a selected group of patients with only limited and resectable peritoneal carcinomatosis. In addition, the trial showed an increased mortality rate. The high mortality rate underlines necessarily strict selection that should be reserved to experienced institutions involved in the management of peritoneal carcinomatosis and gastric surgery. The current PREVENT (FLOT9) - trial is only performed at selected high- volume HIPEC- centers with an expertise in the field.

Based on the negative data for HIPEC- protocols based on intraperitoneal oxaliplatin [13, 14] and the positive data for cisplatin [12], there is a strong rationale for the use of cisplatin instead of oxaliplatin in new HIPEC- protocols, e.g. in our protocol of PREVENT (FLOT9).

We believe that there is a strong theoretical rationale for the conduct of a randomized study evaluating the efficacy and safety of preventive intraperitoneal cisplatin based HIPEC in combination with systemic upfront FLOT- regimen (currently the most effective chemotherapy backbone protocol) in the perioperative treatment of patients with resectable adenocarcinoma with a diffuse or mixed type according to Lauren’s- classification of the stomach or GEJ Type II/III without signs of a systemic, especially a peritoneal seeding.

## Methods/design

### Protocol overview

The PREVENT (FLOT9) study is a multicenter, randomized, controlled and open-label study including patients with localized and locally advanced adenocarcinoma of the stomach and type II/III GEJ of diffuse or mixed type according to Lauren’s classification, scheduled to receive perioperative chemotherapy combined with or without intraoperative HIPEC procedure.

The scope of the trial is to evaluate the efficacy as well as the safety and tolerability of the combination of perioperative chemotherapy with an intraoperative HIPEC for resectable diffuse or mixed type gastric and GEJ (types II/III) adenocarcinoma.

Patients with localized and locally advanced diffuse or mixed type adenocarcinoma of the stomach and type II/III GEJ (i.e. ≥cT3 any N or any T N-positive) with laparoscopic exclusion of peritoneal seeding and radiological exclusion of other distant metastases will be included in this trial after a central review by medical and surgical oncologist.

All enrolled patients will have received 3–6 pre-operative cycles (de-escalation or dose modification allowed) of biweekly FLOT (Docetaxel 50 mg/m^2^ in 250 ml NaCl 0.9%, iv over 1 h; Oxaliplatin 85 mg/m^2^ in 500 ml G5%, iv over 2 h; Leucovorin 200 mg/m^2^ in 250 ml NaCl 0.9%, iv over 30 min; 5-FU 2600 mg/m^2^, iv over 24 h, q2wk) in the preoperative treatment phase. After completion of neoadjuvant FLOT- therapy followed by pre-operative tumor assessment, patients without disease progression (expected to be approximately 90% of the patients) will be included into the trial, stratified by study site, histology type of tumor (Lauren classification diffuse vs. mixed) and initial clinical stage (N- vs. N+). Pts. will be randomized 1:1 to receive either standard of care (SOC) surgery plus postoperative FLOT- regimen (Arm A- standard) or SOC- surgery combined with intraoperative cisplatin based HIPEC- procedure followed by postoperative FLOT- therapy (Arm B- experimental). Randomization will be performed electronically in the eCRF by the site staff using variance minimization, so the sequence of randomization results is not known to the investigators.

### Arm A (FLOT- standard arm)

Surgery in Arm A is planned to occur 4 to 6 weeks after d1 of last FLOT cycle. Surgery is carried out in kind of standardized gastrectomy or transhiatal extended gastrectomy, both including D2- lymphadenectomy. Patients will receive 4 additional post-operative cycles (8 weeks) of FLOT (Docetaxel 50 mg/m^2^ in 250 ml NaCl 0.9%, iv over 1 h; Oxaliplatin 85 mg/m^2^ in 500 ml G5%, iv over 2 h; Leucovorin 200 mg/m^2^ in 250 ml NaCl 0.9%, iv over 30 min; 5-FU 2600 mg/m^2^, iv over 24 h, q2wk) in the post-operative treatment phase. Post-operative treatment should start 6 to 8 weeks, but at maximum 12 weeks, after surgery.

### Arm B (FLOT/ HIPEC- experimental arm)

Surgery in Arm B is planned to occur 4 to 6 weeks after d1 of last FLOT- cycle. Surgery is carried out in kind of gastrectomy or transhiatal extended gastrectomy, both including D2- lymphadenectomy. Surgery will be combined with an intraoperative Hyperthermic Intraperitoneal Chemoperfusion (HIPEC).

HIPEC itself can be performed in closed or open-abdomen procedure, according to the local standards at study site. The protocols advices the prevention of nephrotoxicity during hyperthermic perfusion with cisplatin. Before hyperthermic perfusion starts, urine production should be more or equal than 1 ml/kg/hr. We recommend the usage of sodium thiosulfate, but the usage depends on the local preferences and standards. At start of hyperthermic perfusion: Sodium thiosulfate: 9 g/m2 in 200 ml distilled water, made isotone with sodium chloride, is to be given IV push over 15–20 min, concurrently at start of hyperthermic infusion of cisplatin. This is to be followed by 12 g/m2 thiosulfate IV continuous infusion over 6 h (the 12 g/m2 should be dissolved in 1 l of distilled water and infused at 167 ml/hr). After positioning of inflow catheter and drains intraabdominal cisplatin solution (75 mg/m2 in NaCl 0.9%) will be administered at a temperature of 42 °C for 90 min. Perfusion with cisplatin at a dose of 75 mg per square meter and at a flow rate of 1 l per minute will then be initiated (with 50% of the dose perfused initially, 25% at 30 min, and 25% at 60 min). The perfusion volume will be adjusted such that the entire abdomen is exposed to the perfusate. The HIPEC procedure takes 120 min in total, including the 90-min perfusion period. To prevent heat trauma to normal tissue the temperature of the silicon drain will not be increased over 42 °C. Post-surgical phase: Urine production should not be less than 1 ml/kg during hyperthermic perfusion and for 3 h following surgery.

Patients will receive 4 additional post-operative cycles (8 weeks) of FLOT in the post-operative treatment phase. Post-operative treatment should start 6 to 8 weeks, but at maximum 12 weeks, after surgery.

In both arms, tumor assessments (CT or MRI) are performed before randomization prior to surgery, and then every 3 months (radiological tumor assessment) thereafter until progression/relapse, death, or end of follow-up. A change from CT into MRI in the follow up period is possible at any time. Also during tumor assessment visits and additionally after surgery blood (EDTA-plasma, serum and whole blood for genomic DNA isolation (ccfDNA)) is collected for later translational research projects.

During treatment, clinical visits (blood cell counts, detection of toxicity) occur prior to every treatment dose. Safety of FLOT/ HIPEC will be monitored continuously by careful monitoring of all adverse events (AEs) and serious adverse events (SAEs) reported.

**Fig. 1:**
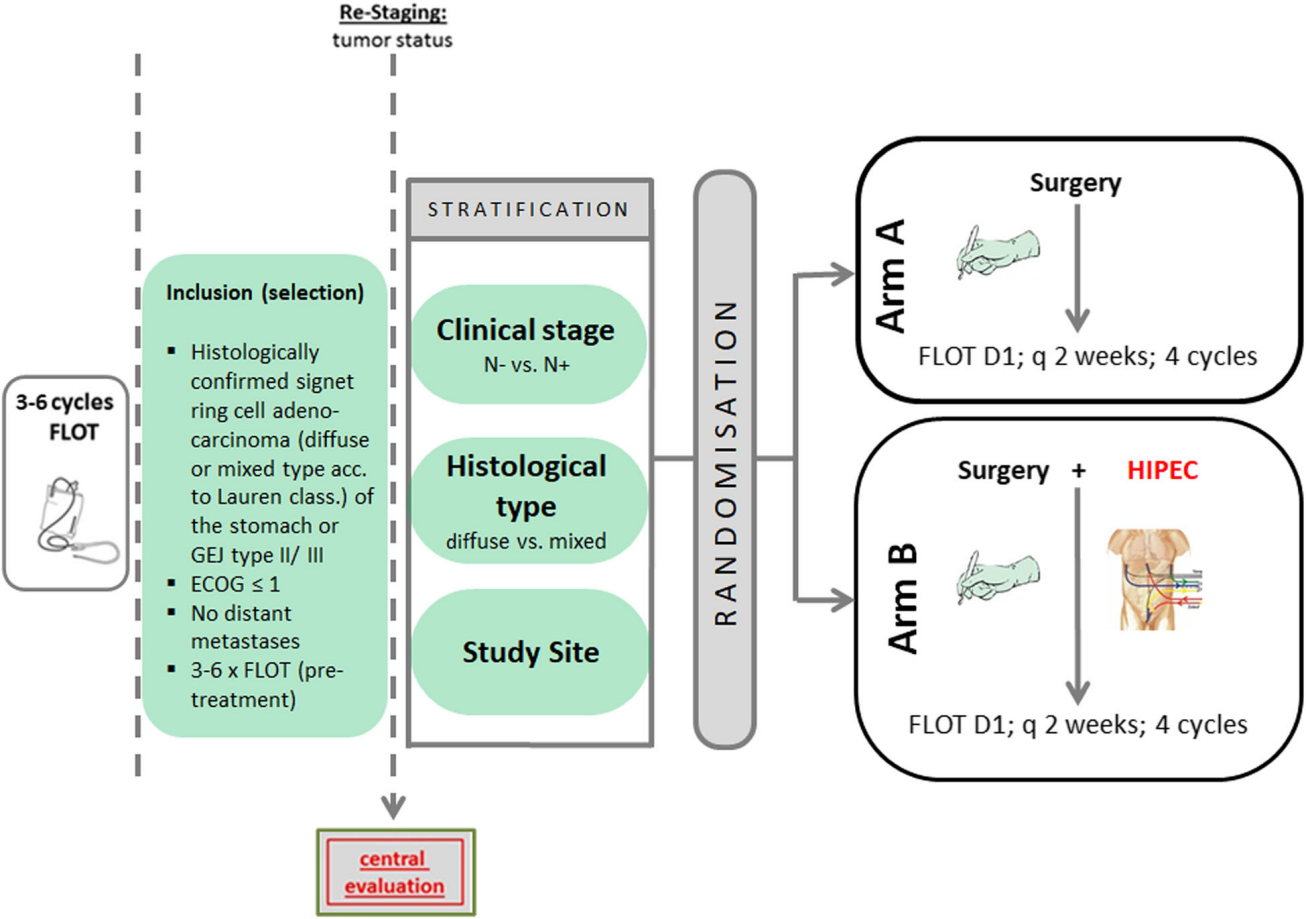
PREVENT (FLOT9) study flow chart (schematic)

The phase III design starts with a safety run-in phase. Eight weeks after 20 patients had curatively intended resection in Arm B, an interim safety analysis is performed that shows feasibility, safety, and tolerability of Arm B planned. It is not planned to discontinue recruitment for the interim safety analysis (see Fig. 1).

### Measures of outcomes and assessments

#### Primary outcome

The primary efficacy objective of the study is to compare progression/disease-free survival (PFS/DFS), defined as first occurrence of progression or recurrence, as determined by the investigator using RECIST 1.1 criteria. The duration of PFS/DFS will be determined by measuring time interval from randomization until disease progression or disease recurrence after surgery or death of any cause.

#### Secondary outcomes

Secondary efficacy objectives are overall survival (OS, defined as the time from randomization to death from any cause), rates of peritoneal relapse at 2 and 3 years in both arms, PFS/DFS rates at 2, 3 and 5 years, OS rates at 3 and 5 years, OS and PFS/DFS (medians and rates) according to subgroups, Quality of life (QoL) – EORTC QLQ C30 and EORTC QLQ STO22 questionnaires, post-operative morbidity/mortality at day 30 after surgery acc. Clavien–Dindo classification, post-operative pain according to Visual analog scale and the safety of perioperative FLOT + HIPEC.

#### Main inclusion criteria

Histologically confirmed, medically operable, resectable diffuse or mixed type adenocarcinoma of the gastroesophageal junction (AEG II-III) or the stomach (uT3, uT4a, any N category, M0), or any T N+ M0 patient is eligible. No prior chemotherapy except 3–6 cycles of FLOT chemotherapy and no prior tumor resection.

#### Main exclusion criteria

Medical inoperability. Inability to understand the study and/or comply with the protocol procedures. Pre-existing peritoneal seeding.

Criteria of primary unresectability, e.g.: radiologically documented evidence of major blood vessel invasion or invasion of adjacent organs (T4b). Patient with involved retroperitoneal (e.g. para-aortal, paracaval or interaortocaval lymph nodes) or mesenterial lymph nodes (distant metastases).

### Treatments

#### Control(s)/comparator(s)

FLOT consists of: Docetaxel 50 mg/m^2^, iv over 2 h, d1; Oxaliplatin 85 mg/m^2^ in 500 ml G5%, iv over 2 h, d1; Leucovorin 200 mg/m^2^ in 250 ml NaCl 0.9%, iv over 1 h, d1; 5-FU 2600 mg/m^2^, iv over 24 h, d1 (= 1 cycle); Start of next cycle on day 15 (every 2 weeks) [7].

#### Dose, mode, and scheme of intervention

In both arms, patients will undergo surgery 4 to 6 weeks after the 3-6th cycle of FLOT.

Surgery is carried out in kind of gastrectomy, transhiatal extended gastrectomy. In the experimental arm surgery will be combined with an intraoperative Hyperthermic IntraPEritoneal Chemoperfusion (HIPEC).

HIPEC itself can be performed in closed or open-abdomen procedure. At start of hyperthermic perfusion, Sodium thiosulfate: 9 g/m^2^ in 200 ml distilled water, made isotone with sodium chloride, is to be given IV push over 15–20 min, concurrently at start of hyperthermic infusion of cisplatin. This is to be followed by 12 g/m^2^ thiosulfate IV continuous infusion over 6 h (the 12 g/m^2^ should be dissolved in 1 l of distilled water and infused at 167 ml/hr). After positioning of inflow catheter and drains intraabdominal cisplatin solution (75 mg/m^2^ in NaCl 0.9%) will be administered at a temperature of 42 °C for 90 min. Perfusion with cisplatin at a dose of 75 mg per square meter and at a flow rate of 1 l per minute will be then initiated (with 50% of the dose perfused initially, 25% at 30 min, and 25% at 60 min). The perfusion volume will be adjusted such that the entire abdomen is exposed to the perfusate. The HIPEC procedure takes 120 min in total, including the 90-min perfusion period. For a more exact description see above under: Arm B (FLOT/ HIPEC- experimental arm).

#### Sample size calculation

The primary efficacy analysis will compare randomized surgical resection combined with HIPEC to randomized surgical resection only on the time to the primary efficacy endpoint using the intent-to-treat population. The hypothesis test will use the log rank test to compare the investigational arms. The study assumes a Hazard ratio of 0.65 favoring the HIPEC group. The PFS/DFS in the reference arm is set as 20.19 months (calculation on complete data from study FLOT4) for signet ring cell containing gastric cancers. Accrual time is 42 months followed by 2 years follow up period. Dropouts prior to randomization are set at 35%. Dropouts after randomization are set 5%. Type I error is 5% and one-sided Log rank test is used. Two hundred patients are to be randomized to provide a statistical power of 80%. These 200 patients will be recruited in 20 German sites with already proven experience in conducting HIPEC procedure.

#### Monitoring

All adverse events and severe adverse events occurring after informed consent are recorded in the patient’s electronic case report form by the responsible site staff. Adverse events will be assessed according to the Common Terminology Criteria for Adverse Events (CTCAE) version 5.0. With this data the safety will be monitored continuously by careful monitoring of all adverse events and serious adverse events reported. A compilation of all serious adverse events is sent to lead Ethic, regulatory body and the Safety Monitoring Board (SMB). The SMB furthermore provides the sponsor with recommendations regarding study modification, continuation or termination. In this process the SMB may give advice for continuation, changes to the study protocol or termination of the study. The SMB may claim unplanned interim analyses of any variable and – beyond the aforementioned items – it may ask for any additional activity within the trial if the activity is on behalf of patients’ security.

Premature termination of the study may also be decided if unexpected severe surgical complications occur, more effective therapies become available or if patient enrollment is insufficient. Final decision is made by sponsor representative and the lead coordinating investigator.

It is understood that an outside monitor and other authorized personnel may contact and visit the investigator, and that they will be allowed direct access to source data/documents for trial-related monitoring, audits, IRB review, and regulatory inspection. Direct access is defined as permission to examine, analyze, verify, and reproduce any records and reports that are important to evaluation of a clinical trial. All reasonable precautions within the constraints of the applicable regulatory requirements to maintain the confidentiality of patients’ identities and sponsor’s proprietary information will be exercised. In case of an audit by the sponsor/sponsor representative or an appropriate authority, the investigator will make all relevant documents available.

#### Ethical considerations, information giving and written informed consent

The study protocol was approved by the responsible lead ethics committee on the July 27th, 2020 under the identification number 2020–1709-fAM. The study has been registered on the ClinicalTrial.gov website under the identification number NCT04447352 and under EudraCT 2017–003832-35. The PREVENT (FLOT9) study complies with the Declaration of Helsinki rules, the principles of Good Clinical Practice guidelines and the Data Protection Act. The trial will also be carried out in compliance to local legal and regulatory requirements. For each patient to be enrolled into the study, obtaining written informed consent prior to inclusion into the study is essential.

## Discussion

Several Asian authors have reported a potential benefit from using intraperitoneal chemotherapy with or without hyperthermia, as an adjuvant therapy following curative surgery [19, 20]. Fujimoto et al. [21] recruited 141 patients and showed that HIPEC significantly reduced the incidence of peritoneal recurrence (*p* < 0.001) and increased the survival rate (*p* = 0.03) without a significant increase in postoperative adverse events. Yonemura et al. [22] showed in a randomized trial of 139 patients favorable 5-year survival rate in gastric cancer patients treated with HIPEC- therapy.

In 2001, Kim and Bae [23] published the results of a controlled study on 103 patients presenting with serosa-positive gastric carcinoma, who underwent surgical resection alone or surgical resection combined with HIPEC with significantly higher 5-year survival rates in the HIPEC group, when stage IV patients were excluded. A meta-analysis by Yan et al. [24] demonstrated that using HIPEC as an adjuvant treatment significantly improved survival rates in stomach cancer and suggested that intraperitoneal chemotherapy + hyperthermia delivered during surgery was a more effective than a delayed approach. The meta-analysis of Coccolini confirmed the potential benefit of using HIPEC for patients with an advanced gastric cancer in an adjuvant setting [25]. However, all the studies mentioned above included mostly patients of Asian origin, where tumor biology, therapeutic strategies, and prognosis differ from those in Western countries. Moreover, the studies had significant shortcomings. The trials were underpowered, endpoints and statistics did not meet the modern quality criteria and the adjuvant chemotherapy regimens are not corresponding to the modern therapies of the western world using FLOT in the perioperative curative or even oligometastatic situation [7, 8, 26].

The potential role of a so called prophylactic HIPEC and even laparoscopic neoadjuvant HIPEC are currently being increasingly used and evaluated. The combination of classic systemic chemotherapy with an intraperitoneal therapy gained popularity already in the last century in the late 90s, because of promising early results in several Phase II trials. Unfortunately, these findings could not be confirmed in e.g. recent trials with intraperitoneal approaches like the PHOENIX-gastric cancer study [27], a randomized controlled trial. The appropriate treatment in gastric cancer with high risk for peritoneal seeding or an already established low burden peritoneal carcinomatosis still remains controversial and a trial in this field of a high unmet need is required.

Peritoneal seeding in advanced gastric cancer patients is detected in up to 30% and associated with a poor prognosis [28, 29] measured by a median overall survival of 3–6 months without treatment and 6–12 months with chemotherapy [30–34].

Classical systemic chemotherapy has only a very limited effect caused by the problem of peritoneal plasma barrier with a reduced permeability of intravenous applicated agents with significantly reduced peritoneal concentration relative to plasma clearance [35], delivering a strong rationale for HIPEC- therapy due to direct delivery of appropriate doses of chemotherapy into the peritoneal cavity [35].

Hyperthermic chemotherapy is able to achieve deeper penetration in the peritoneal cavity and is able to enhance the antitumoral effects of chemotherapy directly at the region of interest [36].

The aim of HIPEC mostly in combination with cytoreductive surgery, is normally to remove all visible peritoneal seedings, while the heated therapy would treat residual microscopic disease.

In our current study population, there are no visible signs of a peritoneal seeding, based on the inclusion criteria and therefore there is no need for cytoreductive surgery but the need for heated chemotherapy to treat potential residual microscopic occult or incidental disease not visibly or cytologically detectable in a high risk group for peritoneal seeding, with the potential to cure. This will be tested in a population of 200 patients. This sample size takes into account the recent FLOT4 data.

A recent meta-analysis by Desiderio et al. [37] of 11 randomized controlled and 21 comparative non- randomized trials was able to show a significant, modest, amelioration in median overall survival with the addition of HIPEC to cytoreductive surgery (CRS) in gastric cancer (HIPEC+CRS vs. CRS, median OS 11.1 vs. 7.1 months, *P* < 0.001). Furthermore lots of individual studies reported that a low volume peritoneal seeding combined with complete cytoreduction are most likely to benefit from HIPEC- therapy, in selected patients [38–40].

In the meta-analysis of Desiderio et al. [37] the addition of HIPEC in patients with locally advanced cT3–4 disease and no evidence of a peritoneal spread demonstrated a decrease in overall disease recurrence (relative risk [RR], 0.73; 95% CI, 0.59–0.89; *P* = 0.002), 3-year (RR, 0.71; 95% CI, 0.53–0.96; *P* = 0.03) and 5-year (RR, 0.82; 95% CI, 0.70–0.96; *P* = 0.01). A meta-analysis by Sun et al. [41] based on 10 randomized trials evaluated the addition of HIPEC to surgery for T4a gastric cancers, with no gross evidence of peritoneal metastatic implants, and reported a significant risk reduction in mortality (RR, 0. 73; 95% CI, 0.64–0.83; *P* < 0.001) as well peritoneal recurrence (RR, 0.45; 95% CI, 0.28–0.72; *P* = 0.001).

The mentioned data provide a strong rationale for a prophylactic HIPEC- approach. The ongoing GASTRICHIP- trial (NCT01882933) [42] is currently recruiting patients with T3/4 gastric tumors, irrespective of nodal or peritoneal cytology status, and randomizing patients to undergo either gastrectomy alone or with HIPEC.

The approach of PREVENT (FLOT9) is even more specific, only recruiting patients with high risk for developing peritoneal metastatic disease, respecting the nature of mixed and diffuse type gastric cancer seeding into the peritoneal cavity compared with the intestinal type where hematogenous spread with a hepatic seeding is more common. Due to respecting the molecular nature of the different gastric cancer types, intestinal type gastric cancers are excluded in our approach, because in future tailored trials it seems to be important not to treat the entire gastric population, but the right pts. to have the possible opportunity for a positive trial with the correct method.

Patients with GC > T2, diffuse or mixed type histological subtype, and lymphovascular invasion are at increased risk of peritoneal relapse, but at this stage still with a potential for cure. However, the benefit of prophylactic HIPEC after radical gastrectomy in these patients remains controversial [43] and needs further evaluation within a randomized trial like the current PREVENT (FLOT9).

## Data Availability

“Not applicable”.

## Abbreviations

ACO: Assoziation chirurgische Onkologie
AIO: Arbeitsgemeinschaft Internistische Onkologie- Working group of Medical Oncologists
CAOGI: Chirurgische Arbeitsgemeinschaft für den Oberen Gastrointestinaltrakt
EORTC: European Organization for Research and Treatment of Cancer
EORTC-QLQ-C30: European Organization for Research and Treatment of Cancer - Quality of Life Questionnaire-C30
EORTC-QLQ STO22: European Organization for Research and Treatment of Cancer - Quality of Life Questionnaire-Stomach 22 questions
FLOT: 5-flourouracil, leucovorin, oxaliplatin, and docetaxel
GEJ: Gastroesophageal junction cancer
QoL: Quality of life
HIPEC: Hyperthermic IntraPEritoneal Chemoperfusion
